# Amelioration of Nephrotoxicity in Mice Induced by Antituberculosis Drugs Using *Ensete ventricosum (Welw.)* Cheesman Corm Extract

**DOI:** 10.1155/2022/6941509

**Published:** 2022-03-17

**Authors:** Abebe Dukessa Dubiwak, Urge Gerema, Daba Abdisa, Ebsa Tofik, Wondu Reta

**Affiliations:** ^1^Division of Medical Biochemistry, Department of Biomedical Sciences, Faculty of Medical Sciences, Institute of Health Sciences, Jimma University, Jimma, Ethiopia; ^2^Division of Clinical Anatomy, Department of Biomedical Sciences, Faculty of Medical Sciences, Institute of Health Sciences, Jimma University, Jimma, Ethiopia; ^3^Department of Biology, College of Natural and Computational Sciences, Mettu University, Metu, Ethiopia; ^4^Division of Medical Physiology, Department of Biomedical Sciences, Faculty of Medical Sciences, Institute of Health Sciences, Jimma University, Jimma, Ethiopia

## Abstract

**Background:**

Antituberculosis drugs are antimicrobial agents important for treating a communicable disease called tuberculosis. Despite their importance, antituberculosis drugs such as isoniazid and rifampicin have severe adverse effects like nephrotoxicity with acute renal failures. *Ensete ventricosum (Welw.)* Cheesman is a nutritional herbaceous perennial plant, and it has indigenous ethnomedicinal values for the society. This study aimed to evaluate the protective role of the *Ensete ventricosum (Welw.)* Cheesman corm extract (EVCE) against nephrotoxicity induced by isoniazid and rifampicin in mice.

**Methods:**

The present study was conducted on thirty Swiss albino mice randomly allocated into five groups. Group-I (only distilled water), Group-II (only isoniazid 75 mg/kg and rifampicin150 mg/kg), Group-III (isoniazid and rifampicin along with 200 mg/kg EVCE), Group-IV (isoniazid and rifampicin along with 400 mg/kg EVCE), and Group-V (isoniazid and rifampicin along with silymarin) were treated for thirty days. At the end of the study, the experimental animals were sacrificed after being injected with anesthetic drug, blood was drawn for a kidney function test, and the kidney was also taken from each experimental animal for histopathological evaluation. Data were entered and analyzed by using one-way ANOVA of SPSS version 25. *Results and Conclusion*. Serum levels of creatinine, blood urea nitrogen (BUN), and uric acid of the Group-II mice were significantly (*P* < 0.01) elevated, and serum levels of total proteins and albumin of Group-II mice were significantly (*P* < 0.01) decreased as compared to Group-I. The group of mice treated with an EVCE reinstated those derangements. The kidney section of Group-II mice showed an abnormality in kidney structure; however, these deformities were not detectable in group-IV mice. The EVCE has sufficient nephroprotective potential against antituberculosis drug-induced kidney injury.

## 1. Introduction

Tuberculosis (TB) is an airborne infectious disease caused by organisms of the *Mycobacterium tuberculosis* complex [1]. TB disease continues to be a profound cause of morbidity and mortality, mostly in low-income and middle-income countries. And it is also the second leading cause of death worldwide due to infectious disease [2, 3].

Today, TB outcomes are worsened with the presence of multidrug-resistant tuberculosis that is mainly due to poor compliance and adherence of patients to the treatment [4]. An adverse effect observed during treatment with antituberculosis drugs was attributed to poor compliance and adherence of TB patients to treatment [5, 6].

One of the severe adverse effects of antituberculosis drugs is nephrotoxicity, which is associated with acute renal failure [7, 8]. Isoniazid (INH) and rifampicin (RIF) are first-line antituberculosis drugs associated with several adverse effects [9]. The most commonly reported side effect of INH and RIF is hepatotoxicity. However, nephrotoxicity such as acute tubular necrosis and interstitial nephritis have also been reported [10].

The kidney is a well-known vulnerable organ to exogenous (therapeutic and environmental xenobiotics) and endogenous toxicants [11, 12]. Nephrotoxicity results from use of various drugs [13], because the kidney is involved in the metabolism of drugs and other xenobiotics [14]. Drug-induced nephrotoxicity remains a significant problem as the use of nephrotoxic drugs is unavoidable in a clinical setting [15].

According to numerous studies, the incidence of RIF-induced kidney damage ranges from 1.8% to 16% of all acute renal failures [16]. The most common laboratory findings in the drug-induced nephrotoxicity are hypoalbuminemia, elevated serum creatinine, serum uric acid level, and blood urea nitrogen (BUN) [17, 18]. *Ensete ventricosum (Welw.)* Cheesman belongs to the Musaceae family, which is a monocotyledonous, monocarpic, herbaceous perennial plant [19] and a source of numerous minerals [19, 20].

In Ethiopia, *Ensete ventricosum (Welw.)* Cheesman is exploited for treating kidney stones, dysuria, liver diseases, expulsion of the placenta, cough, dysentery, healing of bone fractures, diabetes, and microbial infections. It also serves as a staple and costaple food [21–24]. Several bioactive constituents and minerals in the extract of corm of the *Ensete* genus have cell-protective effect and enhance the antioxidant defense system (AODS) of cells by scavenging free radicals [20, 25–28]. Nonetheless, no study has been done on its antinephrotoxic activity. Thus, this study is aimed at evaluating the protective role of the *Ensete ventricosum (Welw.)* Cheesman corm extract (EVCE) against nephrotoxicity induced by INH and RIF.

## 2. Materials and Methods

### 2.1. Chemicals

The INH and RIF were obtained from the pharmacy department of Jimma University, while silymarin was obtained from the Ethiopian Public Health Institute.

### 2.2. Plant Materials

The corm of *Ensete ventricosum* (*Welw.*) Cheesman was obtained from the Oromia region, Wanci woreda, and authenticated by a botanist at the Addis Ababa University National Herbarium. It was air-dried in a shaded area at room temperature and powdered into a coarse powder with the help of a pestle and mortar. The powder passed through a mesh sieve, subsequently soaked in 80% of methanol, and shaken three times per day for three consecutive days for maceration. The extract was filtered with Whatman filter paper, and then the methanol solution was evaporated using a rotary evaporator; eventually, the extract become solid consistency repeatedly lyophilized by a freeze dryer.

Finally, the crude was placed in an airtight container with proper labels and kept in a refrigerator until the experiment commenced.

### 2.3. Preliminary Phytochemical Screening

The crude extracts were tested for the presence of secondary metabolites like alkaloids, terpenoid, flavonoid, phenol, steroid, quinone, saponin, tannin, and glycoside by procedures of previous studies [29–31].

### 2.4. Acute Oral Toxicity Test

The acute oral toxicity tests of the 80% methanol crude extract of EVCE were conducted according to the Organization for Economic Cooperation and Development (OECD) guidelines [32] for testing chemicals. Toxicity was not detected in up to 2000 mg/kg of EVCE.

### 2.5. Doses for Inducing Nephrotoxicity

The drug dose was 75 mg/kg of INH and 150 mg/kg of RIF administered to experimental animals per oral (PO) to induce nephrotoxicity as was used by previous studies [33, 34].

### 2.6. Experimental Animals and Treatment Protocol

A total of thirty male Swiss albino mice, of ages and weights 8–10 weeks and 30 to 40 g, respectively, were obtained from the Jimma University Tropical and Infectious Disease Research Center (JUTIDRC), Jimma, Ethiopia. Subsequently, the experimental animals were allowed to acclimatize to Jimma University postgraduate Veterinary Medicine (JUCAVM) laboratory conditions for two weeks. The experimental animals had access to food (pellets) and tap water at all times (*ad libitum*). They maintained a 12 hr light/dark cycle at an ambient temperature (20–25°C) [35].

At the end of the acclimatization period, the experimental animals were randomly grouped into five groups each consisting of six mice, and they were treated as shown in Table 1.

### 2.7. Body Weight and Relative Kidney Weight

The body weight of the mice was measured weekly to identify body weight changes in all groups of experimental animals. The relative kidney weight of each mouse was calculated as the kidney weight of the mouse divided by its respective body weight multiplied by 100 (g/g).

### 2.8. Blood Collection and Biochemical Analysis

At the end of the experiment, each mouse was injected with 100 mg/kg ketamine/12.5 mg/kg xylazine for intraperitoneal (IP) anesthesia. Then, the blood samples were collected through cardiac puncture and kept at room temperature for 30 min allowed to clot and centrifuged at 3000 rpm for 15 min to separate serum. The separated serum was used for the following biochemical analysis to assess kidney functions by using commercially available kits: serum creatinine (Erba Mannheim, Ltd., India), blood urea nitrogen (BUN) (Erba Mannheim, Ltd., India), serum uric acid (Erba Mannheim, Ltd., India), serum albumin, and serum total protein. All biochemical estimation was performed according to the manufacturers' instructions.

### 2.9. Histopathological Study

Each experimental animal was euthanized by cervical dislocation immediately after blood collection. This procedure was followed by dissection through the neck to the pubis to open the peritoneum cavity to remove the kidney and fix it in 10% buffered formalin. Afterward, the biopsies were dehydrated in an ascending series of ethanol (70%, 80%, 95%, and 100%), cleared in xylene, and embedded in paraffin wax to form tissue blocks. This process was performed overnight with an open tissue processor (LeicaTP 1020, Germany). The tissue blocks were cut at 5 *μ*m thick sections with a ribbon microtome (Leica Model: TP 1020, Germany), mounted on glass slides, and stained with hematoxylin and eosin (H & E). The prepared slides were examined under light microscopy (Olympus CX21FS1, Philippines) with a 40X objective.

### 2.10. Statistical Analysis

Statistical analysis was performed by SPSS version 25, and all statistical comparisons were made by the one-way ANOVA test followed by Tukey's test post hoc analysis. To evaluate initial and final body weight change within the group, a paired *t*-test was used. The results were expressed as mean (*μ*) ±standard error (SE), and a *P* value <0.05 was considered statistically significant.

### 2.11. Ethical Approval

This study was conducted based on the ethics approval letter obtained from the Research and Ethics Review Committee of Jimma University Health Institute. The experimental animals' handling was according to the international guidelines for the care and use of laboratory animals.

## 3. Results

### 3.1. Result of the Preliminary Phytochemical Screening Test

The qualitative phytochemical screening of crude extracts was performed for the identification of the presence of secondary metabolites listed in Table 2.

### 3.2. Body Weights of Swiss Albino Mice

A paired *t*-test was used to compare the initial and final body weight changes of the mice. The final body weight of Group-I and Group-V mice increased significantly when compared with their initial body weight of the group (*P* < 0.01). Similarly, the final body weight of Group-III and Group-IV mice were increased slightly when compared with their initial body weight but was not significant (*P* > 0.05). However, the final body weight of Group-II mice was decreased as compared to their initial body weight which was statistically significant (*P* < 0.05) (Table 3).

### 3.3. Effect of EVCE on Serum Creatinine, BUN, Serum Uric Acid, BUN to Serum Creatinine Ratio, Serum Albumin, and Serum Total Protein

**Figure 1:**
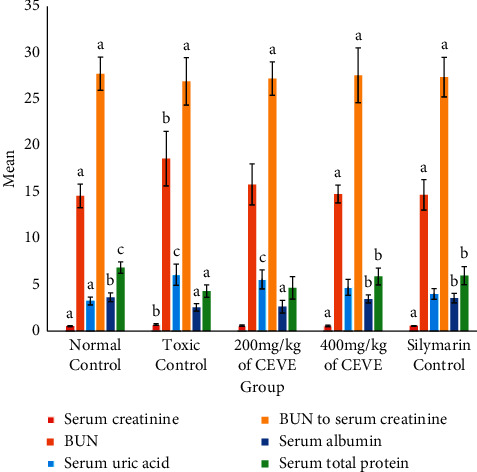
Serum creatinine, BUN, uric acid, BUN to serum creatinine ratio, serum albumin, and total protein levels of experimental animals. The results were expressed as mean ± standard error (SE). Values with different letters (superscript) within the same color bar across different groups are statistically significant (*P* < 0.05), i.e., “a” superscript-containing groups are statistically significantly different as compared to “b” and “c” superscripts-containing groups within the same color bar across different groups.

As illustrated in Figure 1, the serum creatinine, BUN, and uric acid of the toxic control group were statistically significant (*P* < 0.01) and increased as compared to the normal control group, whereas the serum albumin and total protein of toxic control group were statistically significant (*P* < 0.01) and decreased as compared to the normal control group (Figure 1). The serum creatinine, BUN, and uric acid of the 400 mg/kg EVCE-treated group (Group-IV) were statistically significant (*P* < 0.01) and decreased as compared to the toxic control group, whereas the serum albumin and total protein of 400 mg/kg EVCE-treated group (Group-IV) were statistically significant (*P* < 0.01) and increased as compared to the toxic control group (Figure 1).

### 3.4. Effect of EVCE on Relative Kidney Weight (Kidney Weight/Body Weight %)

The relative kidney weight of the toxic control group was statistically significant (*P* < 0.01) and increased as compared to the normal control group (Table 4). However, the relative kidney weight of the extract-treated group (Group-III and -IV) and silymarin-treated group (Group-V) decreased as compared to the toxic control group (Table 4).

### 3.5. Effect of EVCE on Kidney Histopathology

**Figure 2:**
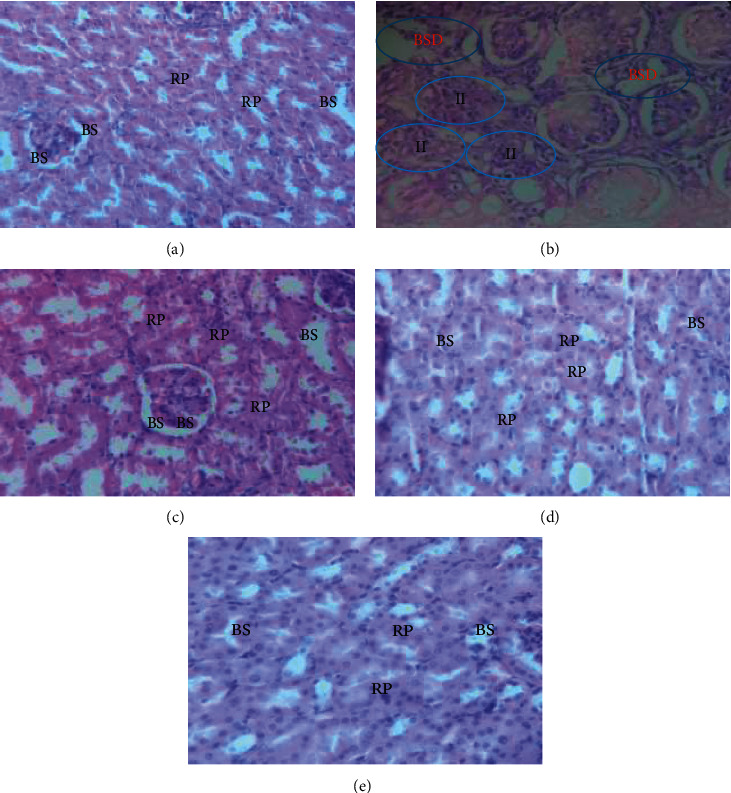
Photomicrograph of the kidney of experimental animals (40X, stained with hematoxylin and eosin): Group-I (a), Group-II (b), Group-III (c), Group-IV (d), and Group-V (e). BSD, Bowman's space dilation; II, inflammatory infiltration; BS, Bowman's space; RP, renal parenchyma.

Microscopic examination of the kidney sections of mice revealed a visible difference in the kidney morphology among the normal controls (Group-I), the toxic control (Group-I), and the treatment groups. The microscopic kidney of the normal control group showed a normal structure of renal parenchyma and normocellular glomeruli. The glomerular capillary loops were easily visible (Figure 2(a)).

In contrast, the kidney section of the INH and RIF only-treated group showed notable alterations: inflammatory infiltration of renal parenchyma (the most prominent findings), the glomerular changes including necrosis in the glomerulus, and patchy lesion. Additionally, renal corpuscles were enlarged with possible mild hypertrophy, and cellular proliferation in the mesangial area was detectable. There was also atrophy of glomerular capillary with noticeable Bowman's space dilation and degeneration of distal and proximal renal tubules (Figure 2(b)).

However, in the group of mice treated with the EVCE, a dose-dependent difference in regenerative capability was observed between Group-III (Figure 2(c)) and Group-IV (Figure 2(d)), significantly reverting the kidney morphology in INH and RIF-treated mice. Furthermore, the silymarin-treated group showed notable restoration of kidney morphology (Figure 2(e)), though minor deformity was detected.

## 4. Discussion

Serum creatinine, BUN, uric acid, and relative kidney weight of the INH and RIF-treated group were statistically significant (*P* < 0.01) and increased as compared to the normal control group (Group-I). The current study shows that clearance of creatinine, urea, and uric acid reduced among the INH and RIF-treated mice; such abnormality might be due to acute kidney injury (AKI) secondary to the formation of immune complexes by antirifampicin antibodies [36]. Furthermore, other studies have reported that reactive oxygen species and oxidative stress play a significant role in the pathogenesis of drug-induced renal damage [3, 37–39]. Thus, nephrotoxic drugs such as antituberculosis render kidney dysfunction like acute tubular necrosis, glomerular and tubulo-interstitial injury, and obstructive nephropathy [40]. The present finding is supported by various studies done at different places and by different investigators [3, 14, 36, 41].

The present study also pointed out that serum total protein and albumin of the INH and RIF-treated group were statistically significant (*P* < 0.01) and decreased as compared to normal control (Group-I). This finding agrees with the studies reported by Shabana et al. [42], Osama et al. [43], Thuawaini et al. [14], Prince et al. [3], and Martin et al. [36] in the INH and RIF-induced rats. This abnormality might be due to antituberculosis drugs inhibiting of protein synthesis [44].

In the EVCE- and silymarin-treated groups (Group-III, -IV, and -V), the serum creatinine, BUN, uric acid levels, and relative kidney weight were decreased. However, statistically significant (*P* < 0.01) decrement was only in the EVCE 400 mg/kg- (Group-IV) and silymarin-treated group (Group-IV) as compared to INH and RIF-treated group (Group-II). This finding was supported by a study done by Sethiya et al. [27] on *Ensete superbum (Roxb.)* Cheesman. Additionally, other studies conducted on the nephroprotective effects of *Ficus religiosa* [7], *Petroslinum crispum*s [41], and *Turmeric* [14] have been consistent with the present finding. This may happen due to cells' protective activities of phytochemicals that are found in EVCE [24].

Moreover, the EVCE-treated groups showed the restoration of total serum proteins and albumin. The total serum proteins and albumin levels were statistically significant (*P* < 0.01) and increased as compared to INH and RIF-treated group (Group-II), which is comparable with the silymarin-treated group. The previous studies done on nephroprotective [36] and antioxidative [3] effects of other plants agree with our finding. This might be due to the nephron being restored and performing its normal activity. It could be also due to the protective activity of EVCE as the result of its active metabolites such as glycosides, alkaloids, flavonoids, tannin, and saponins [45–47].

Furthermore, in the present study, microscopic examination of the kidneys of mice showed a visible difference in the kidney morphology between the controls and the treatment groups (Figures 2(a)–2(e)). The most prominent findings in the kidney section of the INH and RIF only-treated group were inflammatory infiltration of renal parenchyma and glomerular changes include necrosis in the glomerulus and patchy lesions. Additionally, renal corpuscles were enlarged with possible mild hypertrophy, and cellular proliferation in the mesangial area was detectable. There was also atrophy of glomerular capillary with noticeable Bowman's space dilation and degeneration of distal and proximal renal tubules (Figure 2(b)), this result in line with prior studies conducted by Muzika et al. [8], Hashmi et al. [7], Ramadan et al. [41], Thuawaini et al. [14] and Prince et al. [3].

Nevertheless, the group of mice treated with EVCE significantly regenerated the kidney architecture in INH and RIF-treated mice, and a dose-dependent difference in regenerative capacity was observed between Group-III (Figure 2(c)) and Group-IV (Figure 2(d)). This is perhaps due to the active constituents of EVCE might have nephroprotective potential. The restored kidney morphology in the EVCE 400 mg/kg-treated group is comparable with the silymarin-treated group (Figure 2(e)).

## 5. Conclusion

Based on the present finding, we concluded that the EVCE has a nephroprotective role; this is perhaps due to their phytochemical constituents such as tannins, saponins, flavonoids, glycosides, quinone, alkaloids, and steroids. The protective role of the EVCE at 400 mg/kg dose is comparable with the protective activity of the silymarin in antituberculosis-induced toxicity.

## Figures and Tables

**Table 1:** Grouping of experimental animals and treatment protocols.

Groups	The administration type, dose, and duration
Group-I (normal control)	Distilled water 1 ml/kg PO daily for 30 days
Group-II (toxic control)	INH 75 mg/kg + RIF 150 mg/kg PO daily for 30 days as nephrotoxic drugs
Group-III (200 mg/kg of EVCE)	INH 75 mg/kg + RIF 150 mg/kg along with EVCE 200 mg/kg PO daily for 30 days
Group-IV (400 mg/kg of EVCE)	INH 75 mg/kg + RIF 150 mg/kg along with EVCE 400 mg/kg PO daily for 30 days
Group-V (silymarin control)	INH 75 mg/kg + RIF 150 mg/kg along with silymarin 100 mg/kg PO daily for 30 days as nephroprotective drug.

EVCE : *Ensete ventricosum* (*Welw*.) Cheesman corm extract.

**Table 2:** The result of preliminary phytochemical screening of the hydro-methanolic extract of the EVCE.

Phytochemical constituent	Result	Phytochemical constituent	Result
Alkaloid	Positive	Quinone	Positive
Terpenoid	Negative	Saponins	Positive
Flavonoid	Positive	Tannin	Positive
Phenol	Negative	Glycosides	Positive
Steroid	Positive		

“Positive” implies the presence of phytochemicals. “Negative” implies the absence of phytochemicals.

**Table 3:** Mean weight difference of the initial and final body weight of the mice.

Groups	Initial body weight	Final body weight	Mean difference ±SD	*P* value
Group-I (normal control)	36.33 ± 4.03	38.50 ± 3.39	−2.17 ± 1.17^a^	0.006
Group-II (toxic control)	37.33 ± 2.16	34.67 ± 2.80	2.67 ± 1.15^a^	0.007
Group-III (200 mg/kg of EVCE)	37.67 ± 2.25	37.67 ± 3.14	0.00 ± 1.26	1.00
Group-IV (400 mg/kg of EVCE)	36.33 ± 2.06	37.17 ± 1.94	−0.83 ± 1.94	0.34
Group-V (silymarin control)	37.17 ± 2.04	38.83 ± 1.72	−1.67 ± 0.52^a^	0.001

The results were expressed as mean (*μ*) ± standard deviation (SD); “a” implies statistical significance (*P* < 0.05).

**Table 4:** Relative kidney weight in different experimental groups of mice.

Groups	Relative kidney weight
Group-I (normal control)	1.256 ± 0.096^a^
Group-II (toxic control)	1.730 ± 0.343^b^
Group-III (200 mg/kg of EVCE)	1.708 ± 0.126^ab^
Group-IV (400 mg/kg of EVCE)	1.637 ± 0.154^ab^
Group-V (silymarin control)	1.477 ± 0.243^a^

The results were expressed as mean ± SD. Values with different superscripts within the same column are statistically significant (*P* < 0.05).

## Data Availability

Data used to support the findings of this study are available from the corresponding author upon request.

## Abbreviations

EVCE: *Ensete ventricosum* (*Welw*.) Cheesman corm extract
INH: Isoniazid
JUCAVM: Jimma University College of Agriculture and Veterinary Medicine
JUTIDRC: Jimma University Tropical and Infectious Disease Research Center
RIF: Rifampicin.
